# Supplementary material for: The interactome of intact mitochondria by cross-linking mass spectrometry provides evidence for coexisting respiratory supercomplexes
Source: Mol Cell Proteomics. 2017 Dec 8;17(2):216–32. doi: 10.1074/mcp.RA117.000470 (PMC5795388; doi:10.1074/mcp.RA117.000470)
Supplement: Supplemental Data [file supp_RA117.000470_133922_0_supp_23978_fzffwf.zip › spectra_annotation/mito_spectra_annotation/151-1-11-1-3-1.pdf]

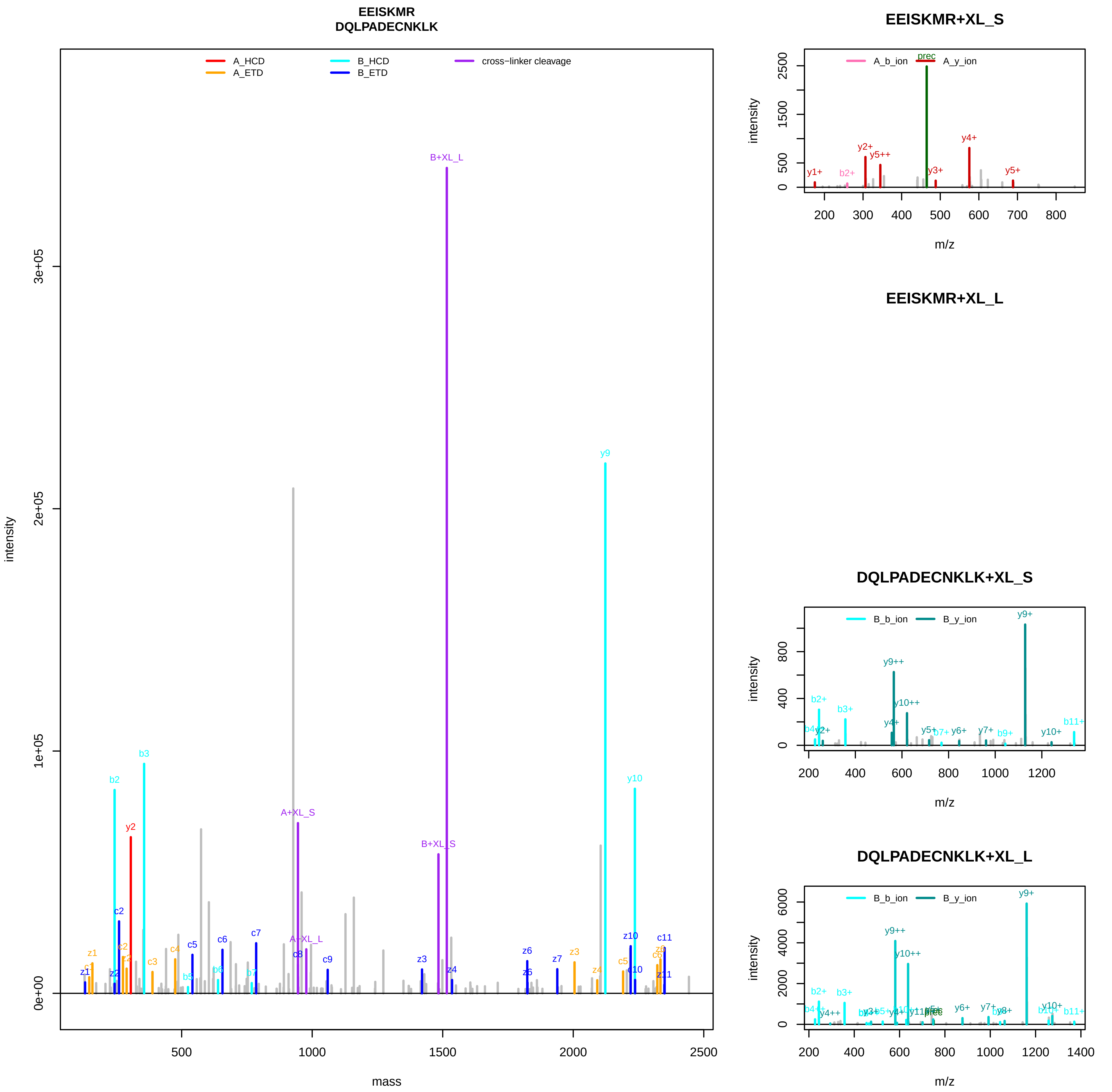

EEISKMR
DQLPADECNKLK
A_HCD
A_ETD
B_HCD
B_ETD
cross−linker cleavage
B+XL_L
y9
A+XL_S
B+XL_S
y10
b2
b3
y2
c2
c5
c6
c7
c9
z6
z7
z10
c11
z1
z2
z3
z4
c3
c4
A+XL_L
intensity
mass
EEISKMR+XL_S
A_b_ion
A_y_ion
prec
y1+
b2+
y2+
y5++
y3+
y4+
y5+
m/z
EEISKMR+XL_L
DQLPADECNKLK+XL_S
B_b_ion
B_y_ion
y9+
y9++
y10++
b2+
b3+
y4+
y5+
y6+
y7+
b9+
y10+
b11+
DQLPADECNKLK+XL_L
